# Decreased fructose-1,6-bisphosphatase-2 expression promotes glycolysis and growth in gastric cancer cells

**DOI:** 10.1186/1476-4598-12-110

**Published:** 2013-09-25

**Authors:** He Li, Juan Wang, Huiyu Xu, Rui Xing, Yuanming Pan, Wenmei Li, Jiantao Cui, Hongbing Zhang, Youyong Lu

**Affiliations:** 1 Laboratory of Molecular Oncology, Key Laboratory of Carcinogenesis and Translational Research (Ministry of Education), Peking University Cancer Hospital/Institute, Beijing 100142, China; 2 State Key Laboratory of Medical Molecular Biology, Department of Physiology and Pathophysiology, Institute of Basic Medical Sciences and School of Basic Medicine, Peking Union Medical College and Chinese Academy of Medical Sciences, Beijing 100005, China

**Keywords:** FBP2, Glycolysis, Gastric cancer, Cell growth, Prognosis

## Abstract

**Background:**

Increasing evidence suggests that cancer is a metabolic disease. Here, we investigated the potential role of fructose-1,6-bisphosphatase-2 (FBP2), the enzyme that catalyses the hydrolysis of fructose-1,6-bisphosphate to fructose-6-phosphate and inorganic phosphate in glucose metabolism, in gastric cancer (GC) development.

**Results:**

Our data indicated that FBP2 was downregulated in GC tissues (86.2%, 100/116), and absent or low FBP2 expression in GC tissues was correlated with poor survival of GC patients (*P* = 0.019). Conversely, ectopic expression of FBP2 in GC cells activated AMP-activated protein kinase (AMPK) signalling, inhibited the Akt-mTOR pathway, suppressed glucose metabolism, enhanced apoptosis, and reduced cell proliferation. Bisulphite genomic sequencing (BGS) in gastric cancer cell lines revealed that the *FBP2* promoter region was densely methylated, and treatment of GC cells with the demethylation reagent, 5-aza-2-deoxycytidine (5-Aza), led to an increase in FBP2 expression. Importantly, forced expression of FBP2 abrogated tumour formation of these GC cells in nude mice.

**Conclusion:**

Our results indicate that FBP2 does negatively regulate cell growth, and reduced expression of FBP2 may contribute to carcinogenesis for GC. These findings suggest that restoration of FBP2 expression can be a promising strategy for the target therapy of GC.

## Background

Gastric cancer (GC), the second most common cause of cancer death worldwide [1], has long been thought to result from a combination of environmental factors and genetic alterations [2]. Activation of oncogenes and inactivation of tumour suppressor genes have all been implicated in gastric tumourigenesis [3,4]. However, increasing evidence in recent years indicates that cancer is a metabolic disease [5], in which cells have lost their normal checks on cell proliferation, resulting in excessive bioenergetic and biosynthetic needs [6]. To sustain such a high demand, cancer cells must alter their metabolism [7]. In the 1920s, Otto Warburg discovered that cancer cells preferentially rely on glycolysis instead of oxidative phosphorylation of glucose in normal cells regardless of the status of oxygen supply [8,9]. As a result, key enzymes in the glycolysis pathway are upregulated to exert this “Warburg effect” for cancer cell proliferation and tumourigenicity [10,11].

Fructose-1,6-bisphosphatase (FBP) is one of the key enzymes in glucose metabolism. This enzyme catalyses the hydrolysis of fructose-1,6-bisphosphate to fructose-6-phosphate and inorganic phosphate and exists as two isoenzymes in mammals: FBP1 and FBP2. FBP1 is recognised as the key enzyme of gluconeogenesis, and upregulated FBP1 has been shown to suppress cancer cell growth [12,13]; recent studies indicate that FBP2 participates in glycogen synthesis from carbohydrate precursors [14].

In this study, we have shown that reduced FBP2 expression was associated with poor clinical outcome in GC patients, while FBP2 upregulation led to inhibition of glucose metabolism, cell proliferation and tumourigenicity. Our findings suggested that FBP2 could be a promising biomarker for predicting the prognosis of GC patients and might provide a potential target for GC therapy.

## Results

### Decreased FBP2 expression in primary tumours is correlated with poor prognosis of GC patients

**Figure 1:**
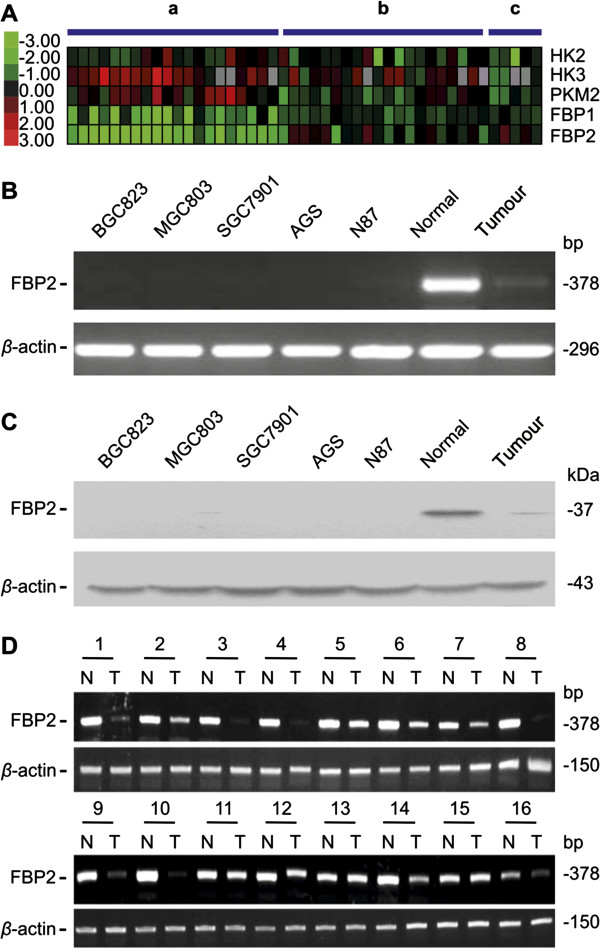
**Decreased FBP2 in GC. (A)** Glycolysis gene alterations were determined using Oligo gene microarray and high-qualified intestinal-type GC tissues between GC (20 cases) and paracancerous (20 cases) and normal gastric tissues (5 cases). Columns represent samples and rows represent genes; red colour represents high expression while green represents low expression. (a) GC versus common reference. (b) Adjacent normal cancer versus common reference. (c) Normal versus common reference. **(B)** Levels of FBP2 mRNA expression in GC cell lines BGC823, MGC803, SGC7901, AGS and N87 by RT-PCR. A pair of GC and adjacent normal tissues was used as controls. **(C)** Levels of FBP2 protein expression in GC cell lines BGC823, MGC803, SGC7901, AGS and N87 by Western blot analysis. A pair of GC and adjacent normal tissues was used as controls. **(D)** Levels of FBP2 mRNA expression in 16 pairs of GC (T) and adjacent normal tissues (N) were determined by RT-PCR.

In our previous study, we used significance analysis of microarrays (SAM) and Bayesian analysis of gene expression levels (BAGEL) to analyse our raw microarray data to identify genes with altered expression in gastric cancer [15]. Among the genes in the glucose metabolism pathway, FBP2 was the most obviously decreased gene in GC samples compared with adjacent and normal tissues (Figure 1A).

To verify the decreased FBP2 expression in GC, the level of FBP2 was determined in both GC cell lines and tissue specimens. FBP2 mRNA was decreased in all 5 GC cell lines (Figure 1B), and downregulation of FBP2 protein was confirmed by Western blot analysis (Figure 1C). RT-PCR analysis represented that decreased FBP2 expression was detected in 88% (14/16) GC tissue specimens compared to adjacent normal tissues (Figure 1D). In addition, immunohistochemistry (IHC) performed in tissue microarrays confirmed that FBP2 protein expression was also decreased in GC tissues, with 86.2% (100/116) of tumour tissues having low FBP2 expression and only 42.7% (47/110) of adjacent normal tissues having low FBP2 expression (Additional file 1: Figure S1, Table 1, *P* < 0.001).

**Table 1:** Analysis of FBP2 expression in gastric tumours

		**FBP2 expression**		
**Histology**	**Cases**	**High (%)**	**Low (%)**	***P***
Normal	110	63 (57.3)	47 (42.7)	**< 0.001**
Tumour	116	16 (13.8)	100 (86.2)	

Bold values indicate P < 0.05.

**Figure 2:**
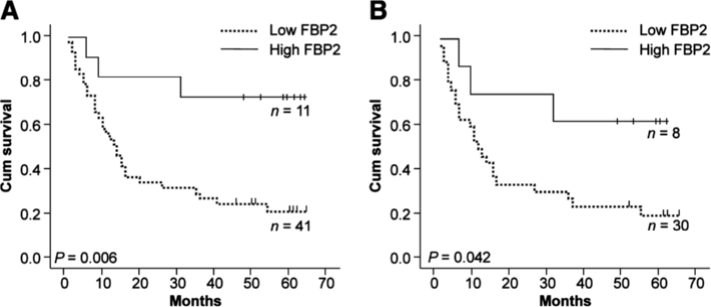
**Kaplan-Meier plots of univariate analysis of overall survival of GC patients according to FBP2 expression of their tumours. (A)** For all the patients, (*n* = 52, *P* = 0.006). **(B)** For the patients with poorly differentiated sample, (*n* = 38, *P* = 0.042).

Kaplan-Meier survival analysis showed that 78.8% (41/52) of patients whose tumours exhibited low FBP2 expression had a poorer outcome than those with high FBP2 expression (Figure 2A, Table 2, *P* = 0.006). More specifically, all the samples were divided into different stages of differentiation. For the patients with poorly differentiated GC samples, those patients with lowly FBP2-expressed samples had poorer outcomes than those with highly FBP2-expressed ones (Figure 2B, *P* = 0.042). Univariate analysis indicated that other factors including tumour depth (*P* = 0.004), lymph node status (*P* = 0.006) and distant metastasis (*P* = 0.020) were also associated with poor survival (Table 2). Multivariate analysis revealed that FBP2 expression was an independent prognostic predictor of GC (Table 3, *P* = 0.019) in conjunction with tumour depth (Table 3, *P* = 0.012).

**Table 2:** Univariate analysis of overall survival

**Variable**	**Cases**	***P***
Gender		
Male	36	0.625
Female	16	
Age		
<60	17	0.496
≥60	35	
Differentiation		
Well/Moderate	14	0.233
Poor	38	
Tumour depth		
T1-T2	12	**0.004**
T3-T4	40	
Lymph node status		
N0	18	**0.006**
N1-3	34	
Distant metastasis		
M0	42	**0.020**
M1	10	
FBP2 expression		
High	11	**0.006**
Low	41	

Bold values indicate P < 0.05.

**Table 3:** Multivariate analysis of overall survival

**Variable**	**Cases**	***P***
Gender		
Male	36	0.478
Female	16	
Age		
<60	17	0.698
≥60	35	
Differentiation		
Well/Moderate	14	0.214
Poor	38	
Tumour depth		
T1-T2	12	**0.012**
T3-T4	40	
Lymph node status		
N0	18	0.088
N1-3	34	
Distant metastasis		
M0	42	0.086
M1	10	
FBP2 expression		
High	11	**0.019**
Low	41	

Bold values indicate P < 0.05.

### FBP2 inhibits cell proliferation and tumourigenicity

**Figure 3:**
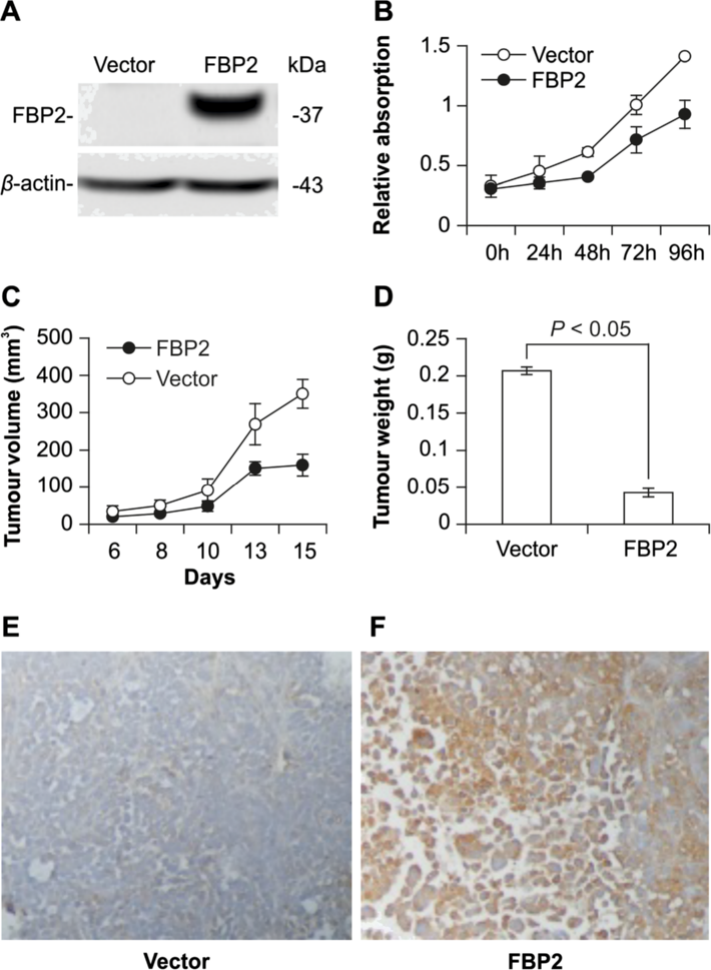
**Functional effects of FBP2 on cell proliferation and tumourigenicity. (A)** Western blot analysis confirmed FBP2 overexpression in transfected BGC823 cells. **(B)** FBP2 overexpression suppressed cell proliferation in the MTT assay (*P* < 0.05). **(C, D)** FBP2-overexpressing BGC823 xenografts exhibited decreased tumour size and weight (*P* < 0.05). **(E, F)** IHC staining showed FBP2 expression status in tumours (200 ×).

To investigate FBP2 function, the BGC823 cell line was transfected with pcDNA3.1-*FBP2* plasmid and used as a cell model for both *in vitro* and *in vivo* studies. Transfection with pcDNA3.1-*FBP2* plasmid resulted in FBP2 overexpression compared with empty pcDNA3.1 plasmid (Figure 3A). The 3-[4,5-dimethylthiazol-2-yl]-2,5-diphenyl tetrazolium bromide (MTT) assay revealed that FBP2 overexpression remarkably reduced cell proliferation in a time-dependent manner (Figure 3B). In addition, BGC823 cells with pcDNA3.1-*FBP2* plasmid formed fewer and smaller colonies than mock transfected cells. More importantly, FBP2 overexpression inhibited the growth of BGC823 xenografts. The combined results from all the mice showed that the tumours formed by BGC823 cells with pcDNA3.1-*FBP2* plasmid were much smaller and weighed less than those formed by mock transfected cells (Figure 3C-D). The clonal origin of BGC823 cells with pcDNA3.1-*FBP2* plasmid in the tumours was confirmed by staining the cells with an anti-FBP2 antibody (Figure 3E-F).

### FBP2 inhibits aerobic glycolysis through interference of Akt-mTOR pathway

**Figure 4:**
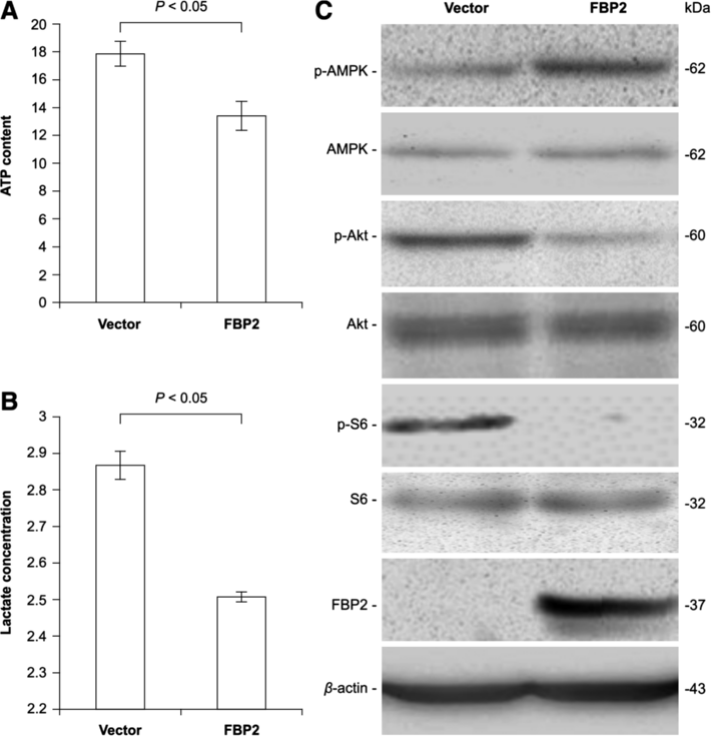
**Functional effects of FBP2 on aerobic glycolysis. (A)** Cellular ATP levels measured using a firefly luciferase-based ATP Assay Kit and normalised to controls showed that FBP2 overexpression inhibited ATP production (*P* < 0.05). **(B)** Lactate levels in cellular culture medium measured using a Lactic Acid Detection Kit and normalised to controls revealed that FBP2 overexpression inhibited lactate production (*P* < 0.05). **(C)** Western blot analysis showed inhibition of the Akt-mTOR pathway in BGC823 cells with pcDNA3.1-*FBP2* plasmid compared to mock transfected cells.

Since FBP2 plays a key role in glucose metabolism [14], the effects of FBP2 overexpression on ATP content and lactate concentration were examined. BGC823 cells with pcDNA3.1-*FBP2* plasmid showed decreased levels of ATP and lactate than mock transfected cells (Figure 4A-B), which decreased the ATP/AMP ratio in these cells. As AMP-activated protein kinase (AMPK) acts as a sensor of cellular energy status and can be activated by a reduction of ATP/AMP ratio [16], the expression of AMPK and other proteins involved in the Akt-mTOR pathway were also examined. The amount of p-AMPK was increased in the BGC823 cells with pcDNA3.1-*FBP2* plasmid. In contrast, the amounts of p-Akt and p-S6 were decreased compared with mock transfected cells, although total AMPK, Akt, and S6 expression remained the same (Figure 4C). These data indicated that FBP2 overexpression led to AMPK activation, and thus inhibited Akt-mTOR signalling.

### FBP2 induces apoptosis

**Figure 5:**
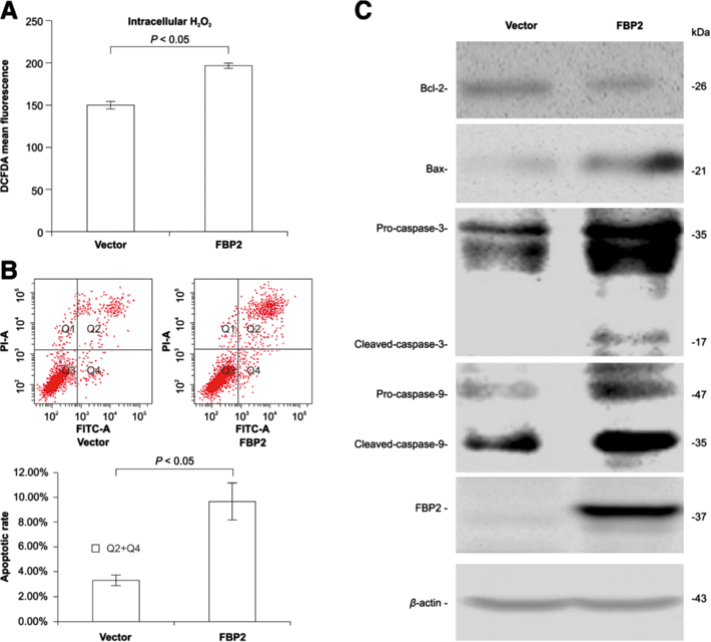
**Functional effects of FBP2 on apoptosis. (A)** FBP2 overexpression increased intracellular ROS determined using a ROS assay kit (*P* < 0.05). **(B)** AnnexinV/PI staining showed that FBP2 overexpression promoted apoptosis in BGC823 cells (*P* < 0.05). **(C)** Western blot analysis showed the effect of FBP2-upregulation on apoptotic-related genes in BGC823 cells.

Since aerobic glycolysis is a protective strategy against reactive oxygen species (ROS) [17] and ROS induces mitochondrial apoptosis [18], the level of intracellular ROS in BGC823 cells with pcDNA3.1-*FBP2* plasmid and the impact of FBP2-overexpression on apoptosis were examined. Intracellular ROS in BGC823 cells with pcDNA3.1-*FBP2* plasmid was higher than that in mock transfected cells (Figure 5A). In addition, annexin V/PI staining showed that BGC823 cells with pcDNA3.1-*FBP2* plasmid had an increased percentage of annexin V^+^/PI^-^ and annexin V^+^/PI^+^ cells, representing early apoptotic cells and late apoptotic/necrotic cells [19], respectively, compared to the mock transfected cells (Figure 5B). Taken together, these data suggested that FBP2 overexpression led to a considerable increase in ROS in apoptotic cells compared to mock transfected cells. Protein profiling of the mitochondrial apoptotic pathway was performed to gain a deeper understanding of the underlying mechanism and revealed that the Bax/Bcl-2 ratio was increased and the activation of their downstream targets, caspase-3 and caspase-9, were all induced in BGC823 cells with pcDNA3.1-*FBP2* plasmid than mock transfected cells (Figure 5C).

### Hypermethylation of *FBP2* promoter reduces the expression of FBP2 in GC

**Figure 6:**
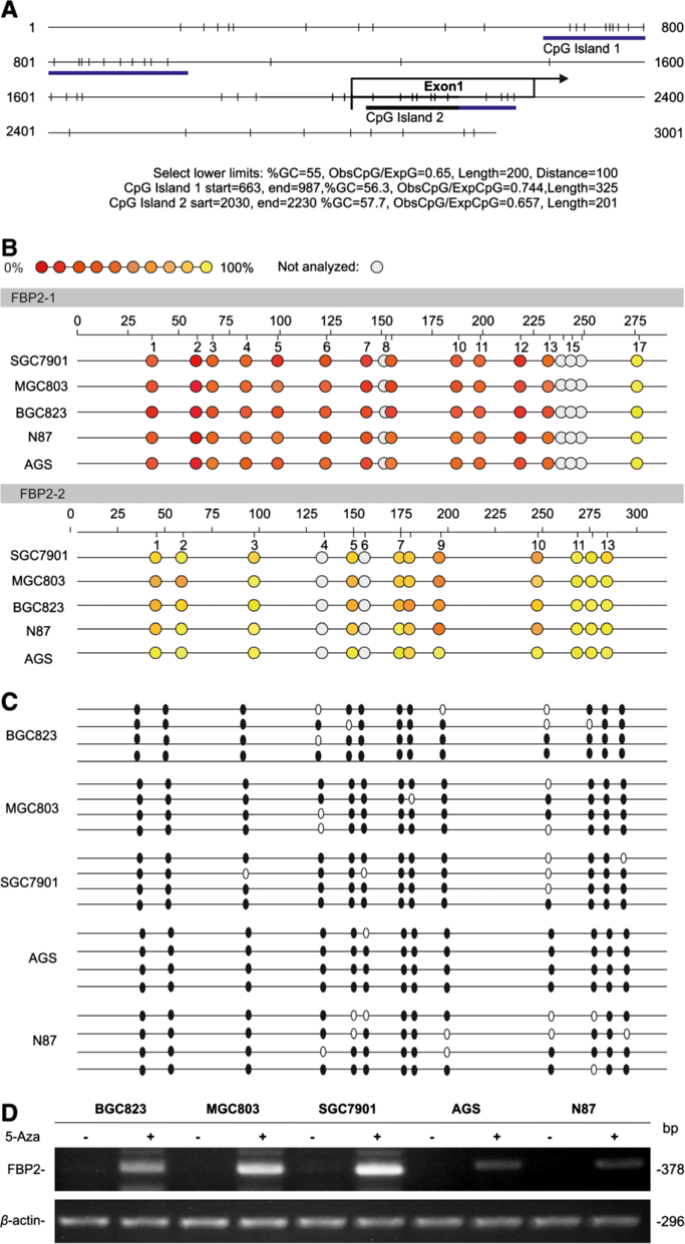
**Decrease FBP2 in GC cells correlated with promoter methylation. (A)** Schematic structure of *FBP2* CpG islands around exon 1 (+1 to +237). **(B)** Profiling of site-specific methylation of *FBP2* CpG islands: *FBP2-1* and *FBP2-2* in 5 GC cell lines: BGC823, MGC803, SGC7901, AGS, and N87. Different colours of circles mark the position of CpG dinucleotides within the sequence (straight line) and the levels of methylation. Gray circles represent the unanalyzed CpG sites. The “not analyzed” CpG sites have low mass or high mass which cannot be reliably detected. Data represent at least three independent experiments. **(C)** Methylation mapping of 13 CpG sites of the *FBP2-2* CpG island obtained from BGS in BGC823, MGC803, SGC7901, AGS, and N87 GC cell lines. White and black circles represent unmethylated and methylated CpG sites, respectively. **(D)** Upregulation of FBP2 mRNA expression following treatment with 5-Aza in GC cell lines.

Methylation status in the genomic *FBP2* promoter sequence (−2000 to +1000 bp) was investigated using the free online software, CpG Island Searcher (GC = 55%; ObsCpG/ExpCpG = 0.65; Length = 200 bp; Gap between adjacent islands = 100 bp) (Figure 6A). Two CpG islands *FBP2-1* (−1338 to −1014 bp) and *FBP2-2* (+29 to +229 bp) were identified and tested for their methylation status using Sequenom’s MassARRAY system in the following GC cell lines: BGC823, MGC803, SGC7901, AGS, and N87. Substantially higher methylation was observed in the *FBP2-2* region than in the *FBP2-1* region, indicating that the *FBP2-2* region may be responsible for the low FBP2 expression observed in the GC specimens (Figure 6B). To confirm this finding, the methylation status of the *FBP2-2* region was determined by bisulphite genomic sequencing (BGS). The *FBP2* promoter was determined to be heavily methylated in BGC823, MGC803, SGC7901, AGS and N87 cell lines using this method (Figure 6C). Importantly, FBP2 expression in these GC cell lines was substantially restored after treatment with the demethylating agent, 5-aza-2-deoxycytidine (5-Aza) (Figure 6D). Furthermore, the FBP2 promoter was densely methylated in GC tissues compared to normal gastric tissues (Additional file 2: Figure S2).

## Discussion

In the present study, we showed that decreased expression of *FBP2* gene in GC tissues correlated with poor patient survival. In addition, tumour depth serves as an important factor of survival for GC patients which shown in our observation. This finding indicated that the metabolic alterations increased the risk of death for GC patients. Conversely, overexpression of FBP2 suppressed glucose metabolism, cell proliferation, and induced apoptosis. Furthermore, CpG methylation of the *FBP2* promoter was correlated with reduction of FBP2 expression, and FBP2 upregulation inhibited the Akt-mTOR and activated mitochondrial apoptotic pathways. Taken together, this inverse correlation between FBP2 expression and cell proliferation suggested that *FBP2* might act as a tumour suppressor gene in GC.

FBP helps to regulate the level of fructose-1,6-bisphosphate, one of the most important intermediates in glycolysis, a major pathway generating ATP from glucose. Glycolysis metabolises glucose to pyruvate, which can then become the precursor of TCA cycle, the other major pathway directing from glucose to ATP. FBP converts fructose-1,6-bisphosphate to fructose-6-phosphate in the reverse direction of glycolysis [20]. Recent studies have shown that FBP1 overexpression could suppress glycolysis in cancer cells [11,12]. Stress that depletes cellular ATP can, in turn, activate energy sensor AMPK [16]. In our study, we found that the levels of ATP and lactate were significantly reduced after FBP2 overexpression, while p-AMPK expression was greatly increased (Figure 4), demonstrating that FBP2 overexpression suppresses energy production by aerobic glycolysis and TCA cycle.

In the present study, FBP2 not only suppressed the glycolysis pathway, but also cell proliferation in GC (Figure 3). Cancer cells face two major metabolic challenges: how to meet the bioenergetic and biosynthetic demands of abnormal proliferation. To overcome these challenges, cancer cells must reprogram their metabolism and become more dependent on aerobic glycolysis [6]. This allows the cancer cells to use the most abundant extracellular nutrient, glucose, to produce abundant ATP. Meanwhile, glucose degradation provides cells with intermediates needed for biosynthetic pathways [21]. These observations explain why the suppression of glycolysis by FBP2 led to inhibition of cell proliferation in GC cells.

The Akt-mTOR pathway, a master regulator of aerobic glycolysis and cellular biosynthesis, plays an important role in satisfying the bioenergetic and biosynthetic needs of cancer cells [21]. The role of Akt-mTOR in cellular energy metabolism is coupled to its function in cell proliferation, and mTOR appears to be the most critical downstream effector of Akt required for cell proliferation and tumourigenicity [22]. Conversely, cellular energy status affects mTOR activity by the negative regulator AMPK [23], which at the same time, has an suppressing effect on Akt activity [24,25]. Thus, FBP2 upregulation in GC cells suppressed aerobic glycolysis, which in turn, inhibited the Akt-mTOR pathway by the negative regulator AMPK (Figure 4), thereby interfering with GC cell proliferation and tumourigenicity (Figure 3).

Since aerobic glycolysis is a protective strategy against ROS [17], the suppression of aerobic glycolysis could lead to an increase in ROS, which has been confirmed to play a role in the alteration of FBP1 [12]. Here, we showed that FBP2 overexpression increased ROS (Figure 5). Moreover, ROS can promote mitochondrial permeabilization and release of cytochrome c, which can stimulate the caspase cascade (e.g. caspase-3, caspase-9), with pro-apoptotic protein Bax and anti-apoptotic protein Bcl-2 as the major players in these pathways [18]. Thus, upregulation of FBP2 induced apoptosis in GC cells by increasing the Bax/Bcl-2 ratio and inducing caspase-3 and caspase-9 activation (Figure 5).

Akt is a major hub of cellular signalling, as its versatile functions can connect diverse upstream signalling pathways to even more diverse physiological outputs, such as promoting cell proliferation and inhibiting apoptosis [26]. In our study, a decrease in Akt could inhibit mTOR downstream targets, and in turn, cell proliferation. In contrast, decreased Akt could also enhance caspases activation in apoptosis. Therefore, FBP2 overexpression can simultaneously link Akt to the mTOR and apoptotic pathways.

Since methylation, one of the major epigenetic modifications of DNA capable of silencing gene transcription, plays an important role in cancer development [27-29], and contributes to the Warburg Effect [30], we also determined the methylation status of *FBP2* promoter. Our findings indicated that the *FBP2* promoter was highly methylated in GC (Figure 6), which has been confirmed in other types of cancer such as brain, liver, breast, and cervical cancers [31]. Thus, the decrease in FBP2 in GC might be correlated with promoter methylation and contribute to carcinogenesis in GC.

## Conclusion

FBP2 overexpression suppresses glucose metabolism and inhibits cell proliferation in GC, while reduced FBP2 expression contributes to cancer development. Taken together, restoration of FBP2 might be a novel and promising target for cancer therapy.

## Methods

### Tissue specimens and IHC

Sixteen specimens of primary GC and their paired noncancerous gastric mucosal tissues were collected from Beijing Cancer Hospital to determine the level of FBP2 mRNA expression. Another 11 specimens of primary GC and 6 normal gastric tissues were collected from Beijing Cancer Hospital to determine the methylation status of *FBP2* promoter. A cohort of 116 formalin-fixed, paraffin-embedded GC tissues was also obtained from Beijing Cancer Hospital, in which 52 cases had follow-up data. This study was approved by the Institutional Ethical Standards Committee with the patients’ written informed consents. The paraffin-embedded microarrays were stained with rabbit anti-FBP2 antibody (Abcam, Cambridge, MA, USA). IHC staining was performed using DAB (DAKO, Carpinteria, CA, USA).

### Cell culture

Human GC cell lines BGC823, MGC803, and SGC7901 were established in China, and the AGS and N87 cell lines were purchased from ATCC (Manassas, VA, USA). All cells were cultured in Dulbecco’s modified Eagle’s medium (DMEM) with 5% foetal bovine serum and incubated in a humidified incubator (5% CO_2_) at 37°C. For demethylation treatment, cells were treated with 5 μM 5-Aza (Sigma-Aldrich, St. Louis, MO, USA) for 72 h. Culture medium and drugs were replenished every 24 h.

### RNA extraction and RT-PCR

Total RNA was extracted from cell lines and tissues using a RNApure total RNA isolation kit (Bioteke Corporation, Beijing, China). Reverse transcription reactions were carried out using EasyScript First-Strand cDNA Synthesis SuperMix (Transgen Biotech, Beijing, China), and semi quantitative RT-PCR was performed using 2 × Taq PCR Mix (Genstar Biosolutions, Beijing, China). The primers used for amplification of FBP2 were 5′-CAGGTTATGCGCTGTACGGT-3′ (Forward) and 5′-TGATGTAGGCCACGGGATTG-3′ (Reverse). *β*-actin was adopted as an internal control in the RT-PCR reactions.

### Sequenom’s MassARRAY EpiTYPER for methylation analysis

The Sequenom MassARRAY platform (CapitalBio, Beijing, China) was used for quantitative analysis of FBP2 methylation. This system employs matrix-assisted laser desorption/ionization time-of-flight mass spectrometry in combination with RNA base-specific cleavage. The primer pairs for amplifying FBP2-1 (−1338 to −1014 bp) and FBP2-2 (+29 to +229 bp) regions were 5′-aggaagagagTTTTTGAGATGGAGTTTTATTTTGT-3′ (FBP2-1 Forward) and 5′-cagtaatacgactcactatagggagaaggctCTCACGCCTATAATCCCAACAC-3′ (FBP2-1 Reverse) and 5′-aggaagagagGAGATTTTGGTAGGGTTAGTATAGATT-3′ (FBP2-2 Forward) and 5′-cagtaatacgactcactatagggagaaggctAAAACTCACAAATAAACCAAACC-3′ (FBP2-2 Reverse). Methylation ratios were generated using EpiTYPER software version 1.0 (Sequenom, San Diego, CA, USA).

### DNA extraction and methylation analysis

Genomic DNA was extracted from cell lines and tissues using the genomic DNA rapid extraction kit (Aurora Biomed, Inc., Vancouver, B.C., Canada). Bisulphite modification of genomic DNA was performed using Zymo DNA Methylation Kit (Zymo Research, Irvine, CA, USA). Methylation status of FBP2-2 region was determined by methylation-specific PCR (MSP) and bisulphite genomic sequencing (BGS). MSP was carried out for 40 cycles with annealing temperature at 61°C. Methylation-specific primers were: MF 5′-ACGGATAGAAGTTTTAGTTCGAAATC-3′ and MR 5′-AATAAACCAAACCGACCTTACG -3′, and unmethylation-specific primers were: UF 5′-TGGATAGAAGTTTTTTTGAAATTGA-3′ and UR 5′-CAAATAAACCAAACCAACCTTACAC-3′. For BGS, the following primers were used: BF 5′-GAGATTTTGGTAGGGTTAGTATAGATT-3′ and BR 5′-AAAACTCACAAATAAACCAAACC-3′. The fragment amplified corresponded to the FBP2 promoter region, +29 and +229 bp, and the ATG start codon of FBP2 was defined as +1. PCR products were inserted into pEASY-T1 Cloning Vector (Transgen Biotech), and at least four colonies were randomly selected for sequencing.

### Ectopic expression of FBP2, cell proliferation and tumour formation

Full-length FBP2 cDNA was cloned into pcDNA3.1 (Invitrogen, Grand Island, NY, USA) to construct the transferring plasmid, pcDNA3.1-FBP2. BGC823 cells were then transfected with either pcDNA3.1-FBP2 or empty pcDNA3.1 plasmid using the Lipofectamine2000 reagent (Invitrogen) and underwent G418 (400 g/mL) selection to form stable colonies. Restoration of FBP2 expression was confirmed by Western blot analysis before these cells were used in any subsequent assays.

Cell proliferation of FBP2-overexpressing BGC823 was assessed using several different assays. For the MTT assay, BGC823 cells with pcDNA3.1-FBP2 or empty pcDNA3.1 plasmid were seeded into 96-well plates, and absorbance at 570 nm was recorded every 24 h for 5 days. For the soft agar assay, BGC823 cells with pcDNA3.1-FBP2 or empty pcDNA3.1 plasmid were suspended in DMEM with 0.3% agarose overlying 0.6% agarose, and colonies were stained with 0.2% INT after 3–4 weeks of culture.

To determine tumour growth *in vivo*, approximately 5 × 10^5^ BGC-823 cells with pcDNA3.1-FBP2 or empty pcDNA3.1 plasmid were injected into female BALB/c nude mice, which were monitored periodically and sacrificed 3 weeks later. Tumour specimens from nude mice were weighed and immunostained. All manipulations involving live mice were approved by the Ethics Committee of Beijing Cancer Hospital/Institute.

### Measurement of ATP and lactate

To measure ATP content and lactate concentration, a total of 2 × 10^5^ cells per well were seeded into 12-well plates for 1 day. Cell numbers were determined using a Countess™ automated cell counter (Invitrogen). Medium was collected to measure lactate levels Nanjing City, China). Lactate levels were determined by recording the absorbance at 530 nm using an Ultraspec 3300 Pro (GE Healthcare Bio-sciences Corp, New Jersey, USA), and data were normalised to the control group. Trypsin-dispersed cells were collected to measure the cellular ATP levels using a firefly luciferase-based ATP Assay Kit (Beyotime, Haimen City, China). Luminescence in the ATP assay was measured using an LMax II luminometre (Molecular Devices, Sunnyvale, CA, USA) and analysed by SoftMax Pro software Version 5 (Molecular Devices). Data were normalised to the control group.

### Measurement of intracellular ROS and annexin V/PI staining

Intracellular ROS was examined using a ROS assay kit (Applygen, Beijing, China), and fluorescence was measured using a BD FACSAria flow cytometer (BD Biosciences, San Jose, CA, USA) (λex at 488 nm and λem at 520 nm). Apoptosis-mediated cell death was examined using the Annexin V-FITC Apoptosis Detection Kit (Beijing Biosea Biotechnology Co. Ltd., Beijing, China). Cells were detected by flow cytometry (BD FACSAria), and data were analysed using FASCDiva software. Cells in early stages of apoptosis were annexin V positive and PI negative, whereas cells in the late stages of apoptosis were both annexin V and PI positive.

### Western blot analysis

Total proteins were extracted from 5 GC cell lines in lysis buffer containing 50 mM Tris (pH 6.8), 100 mM DTT, 1% SDS and 25% glycerol. Equal amount of proteins were electrophoresed on 12% SDS-PAGE and transferred to PVDF membranes using the Mini PROTEAN 3 system (BIO-RAD, Hercules, CA, USA). PVDF membranes were blocked in 5% BSA and incubated with anti-FBP2 (1:1000, Abcam), anti-p-AMPK (1:500, Cell Signalling Technology, Danvers, MA, USA), anti-AMPK (1:500, Cell Signalling Technology), anti-p-Akt (1:500, Cell Signalling Technology), anti-Akt (1:500, Santa Cruz Biotechnology, Inc., Santa Cruz, CA, USA), anti-p-S6 (1:500, Epitomics, Burlingame, CA, USA), anti-S6 (1:500, Cell Signalling Technology), anti-Bcl-2 (1:500, US Biological, Boston, MA, USA), anti-Bax (1:500, Epitomics), anti-caspase-3 (1:250, Bioworld, St. Louis Park, MN, USA), or anti-caspase-9 (1:250, Bioworld) antibody, followed by the appropriate horseradish peroxidase-linked secondary antibody purchased from Proteintech Group Inc. (Chicago, IL, USA). Anti-*β*-actin (1:10000, Sigma) antibody was used to detect the loading control.

### Statistical analysis

All statistical analyses were performed using SPSS 13.0 software. Student’s t test was used to compare groups in the MTT and soft agar assays. Student’s t test was also used to determine remarkable differences between groups regarding tumour size and weight, ATP content and lactate concentration, ROS and apoptotic rate. The χ^2^ and Fisher’s exact test were used to determine the difference of FBP2 protein expression levels between GC and adjacent normal tissues. The Kaplan-Meier method was used for plotting survival curves, and survival data were evaluated using multivariate Cox regression analysis. For all analyses, data were shown as mean ± SD, and *P* < 0.05 was considered statistically significant.

## Abbreviations

GC: Gastric cancer; FBP: Fructose-1,6-bisphosphatase; IHC: Immunohistochemistry; DMEM: Dulbecco’s modified Eagle’s medium; 5-Aza: 5-aza-2-deoxycytidine; MSP: Methylation-specific PCR; BGS: Bisulphite genomic sequencing; MTT: 3-[4,5-dimethylthiazol-2-yl]-2,5-diphenyl tetrazolium bromide; ROS: Reactive oxygen species; AMPK: AMP-activated protein kinase.

## Competing interests

The authors declare that they have no competing interests.

## Authors’ contributions

HL conceived the study, carried out the experiments, analysed data and drafted the manuscript; JW, HYX, YMP, WML and JTC carried out the experiments; RX conceived the study and analysed data; HBZ reviewed the manuscript and supervised the study; and YYL conceived and supervised the study, analysed data and finalised the manuscript. All authors read and approved the final manuscript.

## Supplementary Material

Additional file 1: Figure S1 Representative immunohistochemical staining of FBP2 in tissue microarrays (original magnification × 200). (A) Normal gastric tissue. (B) The cervical part of normal gastric tissue. (C) Well/moderately differentiated GC tissue with high FBP2 expression. (D) Well/moderately differentiated GC tissue with low FBP2 expression. (E) Poorly differentiated GC tissue with high FBP2 expression. (F) Poorly differentiated GC tissue with low FBP2 expression. (G) A transition staining of FBP2 from GC to adjacent normal tissue. Click here for file

Additional file 2: Figure S2 The methylation status of FBP2 promoter was determined by MSP and USP (unmethylation-specific PCR) in GC (T) and normal gastric tissues (N). Primer efficiency was verified by positive control (*in vitro* methylated DNA, IVD) and negative control (normal lymphocyte DNA, NL). ddH_2_O, double-distilled water, was used as blank control. M, methylated alleles; U, unmethylated alleles. Click here for file
