# Supplementary figures and images for: Decreased fructose-1,6-bisphosphatase-2 expression promotes glycolysis and growth in gastric cancer cells
Source: Mol Cancer. 2013 Sep 25;12:110. doi: 10.1186/1476-4598-12-110 (PMC3849177; doi:10.1186/1476-4598-12-110)

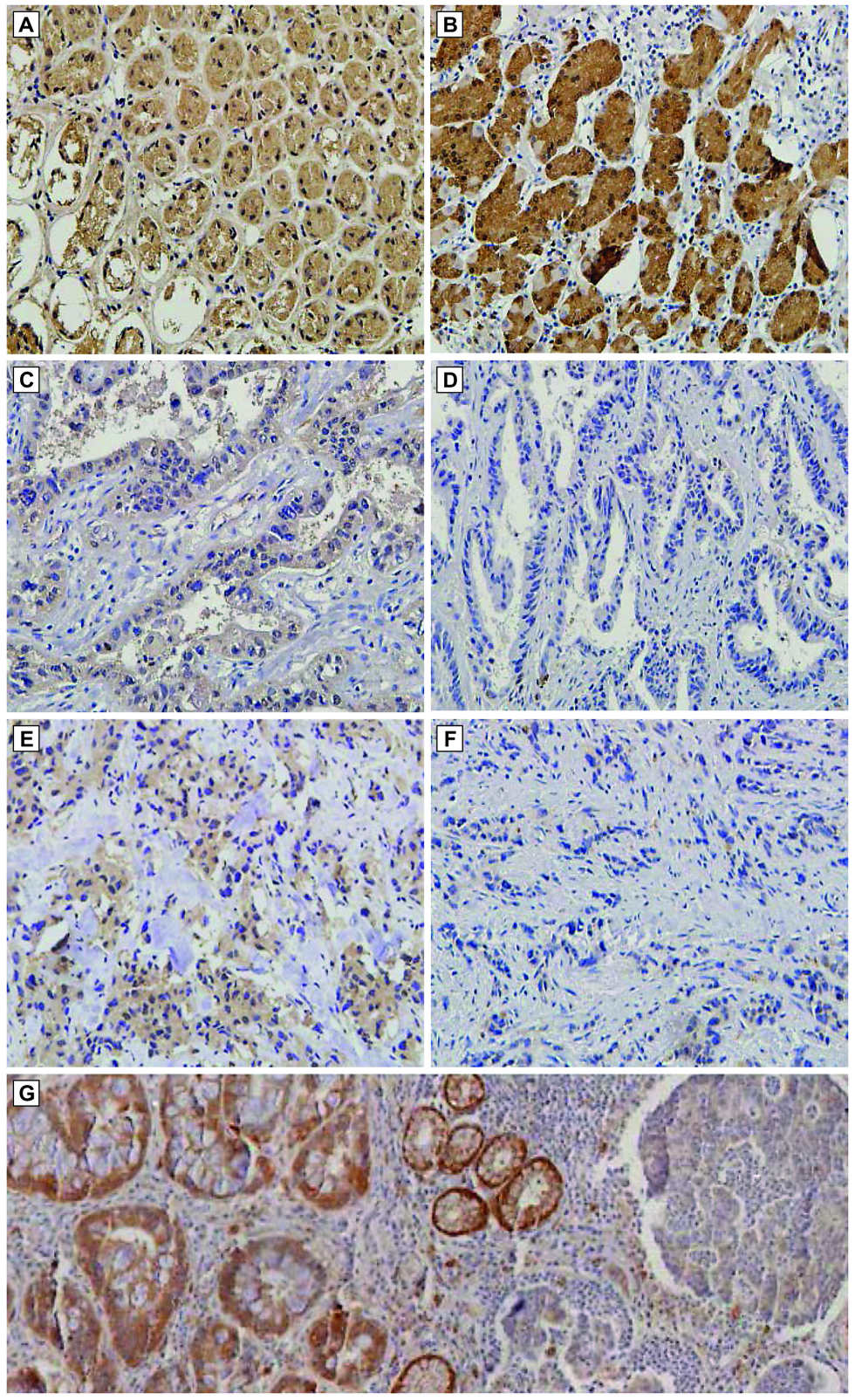

Supplement: Additional file 1: Figure S1 — Representative immunohistochemical staining of FBP2 in tissue microarrays (original magnification × 200). (A) Normal gastric tissue. (B) The cervical part of normal gastric tissue. (C) Well/moderately differentiated GC tissue with high FBP2 expression. (D) Well/moderately differentiated GC tissue with low FBP2 expression. (E) Poorly differentiated GC tissue with high FBP2 expression. (F) Poorly differentiated GC tissue with low FBP2 expression. (G) A transition staining of FBP2 from GC to adjacent normal tissue. [file 1476-4598-12-110-S1.tiff]

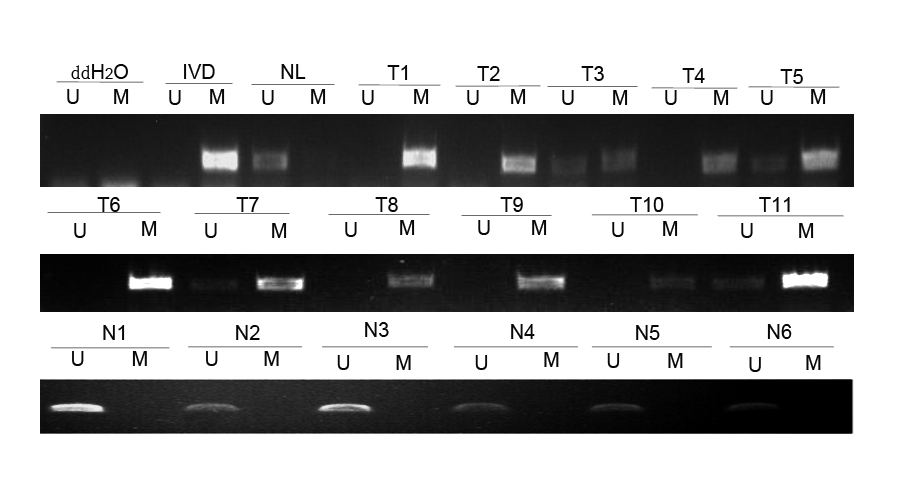

Supplement: Additional file 2: Figure S2 — The methylation status of FBP2 promoter was determined by MSP and USP (unmethylation-specific PCR) in GC (T) and normal gastric tissues (N). Primer efficiency was verified by positive control (in vitro methylated DNA, IVD) and negative control (normal lymphocyte DNA, NL). ddH2O, double-distilled water, was used as blank control. M, methylated alleles; U, unmethylated alleles. [file 1476-4598-12-110-S2.tiff]
